# Use of methotrexate in the treatment of ectopic pregnancies: a retrospective single center study

**Published:** 2020-03-27

**Authors:** C Beguin, G Brichant, L De Landsheere, L Tebache, S Karampelas, L Seidel, M Nisolle

**Affiliations:** University of Liege, 4000 Liege, Belgium;; Obstetrics and Gynecology Department, University of Liege, 4000 Liege, Belgium;; Obstetrics and Gynecology Department, 4000 Liege, Belgium;; Biostatistics, University Hospital of Liege, 4000 Liege, Belgium.

**Keywords:** ectopic pregnancy, pregnancy of unknown location, medical treatment, methotrexate, hCG threshold, predictive factors

## Abstract

**Introduction:**

The aim of this study was to evaluate the efficacy of methotrexate (MTX) in the treatment of ectopic pregnancies. We identified predictive factors of success or failure and compared our results with previous studies to make recommendations for its use.

**Material and methods:**

A cohort of 61 patients from a single center was retrospectively analyzed. Inclusion criteria were a diagnosis of ectopic pregnancy and treatment with a single-dose injection of MTX. The need to perform surgery despite MTX was defined as treatment failure while needing a second MTX injection was not.

**Results:**

In our cohort, MTX demonstrated a success rate of 80%. This rate rose to 84% when patients with human Chorionic Gonadotropin (hCG ) > 5,000 IU/L were excluded. Twenty percent underwent surgery for pain, increased mass size and/or suboptimal hCG kinetics. Low hCG levels on days 0, 4 and 7 as well as the absence of pain, metrorrhagia and hemoperitoneum were predictive of success. MTX was also efficient in the treatment of persisting pregnancies of unknown location (PUL).

**Conclusion:**

Our results are consistent with previous studies and emphasize the fact that MTX is less effective above a certain level of hCG. We obtained a cut-off value of 2439 IU/L with a sensitivity of 66.7% and a specificity of 93.9%. MTX should not be used when hCG is higher than 5,000 IU/L and laparoscopic surgery should be performed. Our results bring additional data about the efficacy of MTX in the management of persisting pregnancies of unknown location.

## Introduction

Ectopic pregnancies (EP) represent about 2% of pregnancies and remain responsible for a significant cause of maternal mortality (Oron and Tulandi, 2013; Bonin et al., 2017). The incidence of EP has increased in developed countries with the emergence of assisted reproduction techniques (Rabischong et al., 2011; Orozco et al., 2015). However, it tends to be diagnosed earlier, which allows for better medical management and the possibility of avoid ing surgery in selected cases (Rabischong et al., 2011; Cecchino et al., 2014; Orozco et al., 2015).

Methotrexate (MTX) has been used in clinical practice as a medical treatment option in these pregnancies since 1982 (Tanaka et al., 1982). However, there is no consensus in the on the human Chorionic Gonadotropin (hCG) threshold above which MTX use is not recommended (Bonin et al., 2017).

The aim of this study is to analyze data from 61 patients who underwent MTX treatment in order to identify predictive factors for its efficacy and make recommendations for its use.

## Materials and methods

### Patients

This retrospective single center study included patients who underwent MTX treatment for an EP (i.e. with a tubal mass or a persisting PUL considered as EP) in the “Centre Hospitalier Régional” (CHR Liège) in Liège, Belgium, between January 2015 and January 2018 inclusive (37 months). Data from 61 patients were collected from the Hospital Pharmacy Registry and digital medical files.

Inclusion criteria were a diagnosis of EP and MTX treatment. Exclusion criteria were MTX treatment for a cornual, cervical or cesarean section scar pregnancy.

All patients underwent a thorough medical history check, clinical examination, transvaginal ultrasound (TVS) and blood sampling including hCG, liver and kidney function and a complete blood count. Hepatic or kidney dysfunction, anemia, thrombocytopenia and/or leucopenia were considered as contra- indications for MTX injection.

In all cases, MTX dosage was 1 mg/kg and the injection was intramuscular, without any local complication. After injection on Day 0 (D0), patients were asked to come back for hCG testing on day 4 (D4) and day 7 (D7). Control with TVS was performed on D7. In case of suboptimal hCG decrease on D7 (i.e. decrease lower than 15% between D4 and D7), a second MTX treatment could be administered. HCG level was then controlled weekly until becoming negative (  2 IU/L).

Evaluation of MTX success was evaluated as described by Bonin et al. (2017), Orozco et al. (2015), Levin et al. (2019) and other studies by considering as success the complete negativation of hCG level after MTX injection and as failure the need to perform surgery (a second MTX injection was not considered as failure). The need for surgery was based on pelvic pain (de novo, persisting or worsening), an increased size of the adnexal mass at TVS and/or suboptimal hCG kinetics. Depending on the condition of the tube during laparoscopy, to the medical and surgical history and to patient preferences, conservative surgery (salpingotomy) or radical surgery (salpingectomy) was performed.

Patient follow-up was analyzed to identify predictive factors of the efficacy of MTX. Data collected included patient age, gestational age, symptoms, characteristics upon TVS and hCG levels on D0, D4 and D7, as well as the number of injections before hCG level became negative and the need for surgery despite medical treatment (Table I). Depending on response, patients were classified in two groups in order to analyze the relevance of predictive factors on the outcome of MTX treatment (Tables II and III).

**Table I:** — Patient characteristics (n=61) (quantitative variables).

Variable	N	Mean	SD	Min	Median	Max
Mean age (years)	61	31	5	21	31	43
Mean gestational age (days of amenorrhea)	44	41	12	7	41.5	67
Ectopic pregnancy diameter at TVS (cm)	24	1.8	0.7	0.4	1.8	3.6
hCG level at D0 (IU/L)	61	1384	1883	60	677	9508
hCG level at D4 (IU/L)	45	1909	3615	33	679	21 962
hCG level at D7 (IU/L)	52	1392	2961	4	298	18 623

**Table II:** — Clinical and biological parameters in the success and failure groups (quantitative data).

MTX treatment	Success	Failure	OR	P value
Maternal age (years)	30.9 ± 5.6 (21-43)	31.2 ± 5.0 (25-39)	1.01	.82
Gestational age (days)	40 ± 13 (7-67)	42 ± 11 (27-57)	1.01	.79
Mass size at diagnosis (cm)	1.9 ± 0.7 (0.6-3.6)	1.6 ± 0.7 (0.4-2.4)	0.57	.41
hCG levels on D0 (IU/L)	793 ± 795 (60-3563)	3,801 ± 2,942 (253-9508)	4.92	.0007
hCG on D0 (IU/L), categories				
< 2000	43 (93.5)	3 (6.5)	1.00	
2000 – 3000	5 (71.4)	2 (28.6)	5.73	
> 3000	1 (12.5)	7 (87.5)	100.3	

**Table III:** — Clinical and imaging data pre- or post-treatment in the success and failure groups (qualitative data).

MTX treatment	Success: n (%)	Failure: n (%)	OR	P value
Total number of patients	49 (80)	12 (20)	/	/
Previous ectopic pregnancy				
Yes	7 (64)	4 (36)	0.33	.20
No	42 (84)	8 (16)		
Conception				
Spontaneous	40 (80)	10 (20)	1.00	.89
Ass. Reprod	9 (82)	2 (18)	0.89	
Symptoms at diagnosis				
Yes	42 (84)	8 (16)	0.33	.14
No	7 (64)	4 (36)	1.00	
Pain at diagnosis				
Yes	31 (79)	8 (21)	1.16	.83
No	18 (82)	4 (18)	1.00	
Metrorrhagia at diagnosis				
Yes	31 (82)	7 (18)	0.81	0.75
No	18 (78)	5 (22)	1.00	
Ultrasound at diagnosis				
Mass	19 (66)	10 (34)	7.90	.013
PUL	30 (94)	2 (6)	1.00	
Fluid in the Douglas pouch at diagnosis				
Yes	18 (82)	4 (18)	0.86	.83
No	31 (79)	8 (21)	1.00	
Pain after MTX				
Yes	13 (54)	11 (46)	18.6	.008
No	22 (96)	1 (4)	1.00	
Metrorrhagia after MTX				
Yes	11 (55)	9 (45)	6.27	.016
No	23 (88)	3 (12)	1.00	
Fluid in the Douglas pouch after MTX				
Yes	6 (37)	10 (63)	22.5	.0005
No	27 (93)	2 (7)	1.00	

### Statistical analyses

Results are presented as means ± standard deviation (SD) or as frequency tables. Normality was tested by the Shapiro-Wilk test and some variables (hCG) were log transformed to be normally distributed. Univariate and multivariate logistic regression was performed to predict failure of MTX and presence of mass according to different variables. A ROC (Receiver Operating Characteristic) and the Youden method were used to calculate a cut-off for the hCG rate. Results were considered significant at the 5% level (P < 0.05). Statistical analyses were performed using GraphPad Prism version 5 (GraphPad Software, San Diego, CA, USA) and with SAS version 9.4 (SAS Institute, Cary, NC, USA).

## Results

Between January 2015 and January 2018, 61 patients received MTX for a tubal EP (48%) or for a persisting pregnancy of unknown location (PUL) (52%). At diagnosis, patients were on average 31 years old and mean gestational age was 41 days. Most patients were symptomatic (82%). Forty-eight percent had a positive ultrasound including 36% with a small hemoperitoneum in the Douglas pouch. Thirty-two patients (52%) were considered as EP even though no pregnancy could be visualized and were classified as persisting PULs. A PUL can be an early intrauterine pregnancy, an ectopic pregnancy or a spontaneous abortion. It refers to cases in which hCG level is higher than 2 IU/L but the location of the pregnancy cannot be found with TVUS (Fields and Hathaway, 2017). A PUL is defined as persisting when the hCG kinetics is suboptimal. In this case, it is more likely to be an EP and it was thus treated as such.

**Figure 1:**
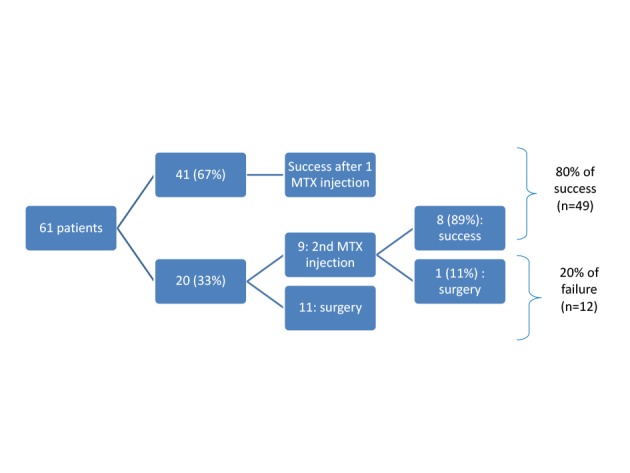
Flowchart of response to Methotrexate treatment. Success was achieved in 80% of women.

A unique MTX injection was successful in 41 women (67%). A second injection was needed in 9 patients for suboptimal hCG decrease. The second injection was successful in 8 of them (89%). The mean hCG level on D0 in patients successfully treated with 1 injection was 758 ± 800 (60 – 3,563) versus 1,025 ± 884 (156 – 2,452) IU/L in those successfully treated with 2 injections (P = .287). The overall success rate was therefore 80% (49/61) (Figure 1).

Surgery was considered as treatment failure and was necessary for 20% of the patients. However, it is worth noting that 3 of these women received MTX even if their D0 hCG level was higher than 5000 IU/L (5222, 8786 and 9508 IU/L, respectively), which is usually not recommended. If these 3 patients are excluded, the success rate reaches 84%.

The mean time to reach a hCG level < 2 IU/L was 25 ± 10 (10 – 44) days in 32 evaluable patients in the success group.

Twelve patients (20%) underwent surgery because of pain after injection (de novo, similar or worsening, 10/12 patients), an increased size of the adnexal mass at TVS (11/12 patients) and/or suboptimal hCG kinetics (8/12 patients). Surgery procedures were as follow: salpingotomy (n=2, 16.7%), salpingectomy (n=8, 66.7%) and hemoperitoneum aspiration with no obvious trophoblastic material (n = 1, 8.3%). In one patient (8.3%), no anomaly was detected during laparoscopy.

The patients were separated in 2 groups according to the success (n=49) or failure (n=12) of MTX treatment (Tables II and III). Various parameters were statistically compared in order to identify potential predictive factors of success or failure.

Maternal age, gestational age and mass size at TVS at diagnosis had no impact on the outcome of MTX treatment. In contrast, hCG levels were highly predictive of treatment failure. The influence of hCG levels was still statistically significant after exclusion of the 3 patients with hCG > 5000 IU/L. Although we lacked data on D4 for 16 women and on D7 for 9 women, mainly because their follow-up was partially carried out away from the hospital, our statistical analyses demonstrate that hCG levels on D4 and D7 can also be considered as predictive factors.

Type of conception, presence of symptoms and fluid in the Douglas pouch at diagnosis did not have a significant influence on the success of MTX treatment.

**Figure 2:**
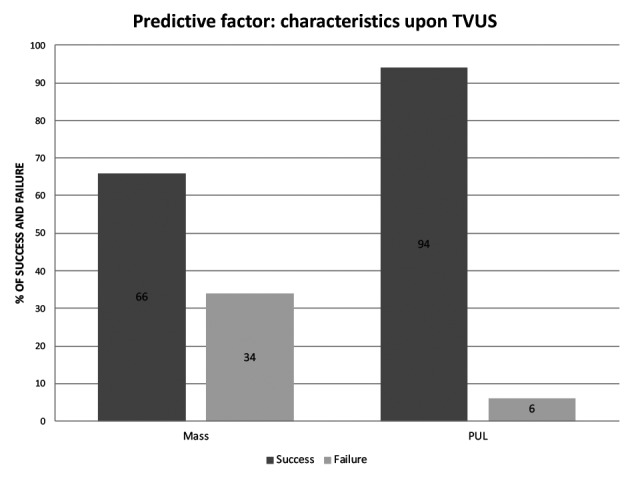
Influence of pre-treatment characteristics upon trans-vaginal ultrasound on the outcome of Methotrexate treatment. The presence of an adnexal mass increases the risk of failure.

However, the presence of an adnexal mass at TVS pre-treatment (Figure 2, OR 7.90), and the presence of pain (OR 18.6), metrorrhagia (OR 6.27) and hemoperitoneum (OR 22.5) post-treatment were predictive factors of failure.

We performed a multivariate analysis with the significant parameters measured before MTX use. With this model, only hCG level on D0 was still a statistically significant predictive factor of MTX treatment outcome.

**Figure 3:**
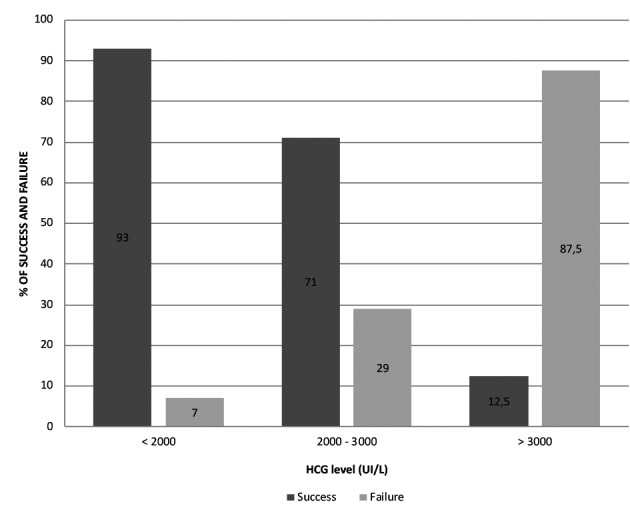
Success and failure rates according to hCG level at Day 0. Chances of success are higher when hCG level is < 2000 IU/L and risks of failure are higher when hCG level is > 3000 IU/L.

The success rate was assessed according to the classification of patients in subgroups depending on hCG levels on D0: < 2000, 2000-3000 or > 3000 IU/L (Figure 3). The treatment in women with hCG > 5000 IU/L failed while it succeeded in virtually all those with hCG < 2000 IU/L. Risk of failure is higher when hCG level on D0 is > 3000 IU/L than when it is < 2000 (OR=100.3). When comparing hCG levels between 2000 and 3000 with levels < 2000 IU/L, the odds ratio is 5.73.

**Figure 4:**
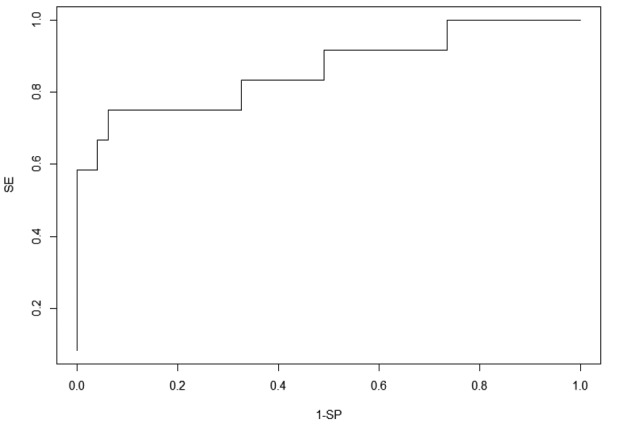
ROC curve for hCG level at Day 0. A cut-off value of 2439 IU/L was obtained with a sensitivity of 66.7% and a specificity of 93.9%.

According to the model with hCG level on D0, we drew a ROC curve (Figure 4). With the Youden method, we reported for each hCG level (log) its sensitivity and specificity to find a precise threshold for MTX use. We obtained for this study the cut- off value of 2439 IU/L on D0 with a sensitivity of 66.7% and specificity of 93.9%.

Lastly, as more than half of our patients presented with a PUL, we compared these patients with those having an adnexal mass (Table IV).

These results emphasize the fact that the incidence of PULs increases with assisted reproduction techniques. It also increases with maternal age but this might be correlated with the higher proportion of assisted reproduction in older women. The likelihood to present with a mass is higher in cases of previous EP. PULs are associated with lower hCG levels, the presence of an adnexal mass seems to be associated with higher levels. In our study, PULs were more frequent when the hCG level was < 2000 IU/L while a hCG level > 3000 IU/L was always associated with an adnexal mass. These results can explain the fact that MTX treatment is very efficient in the management of PULs as we showed that the chance of success is higher with low hCG levels. The presence of symptoms is not statistically correlated to the presence of a mass but the likelihood of having a hemoperitoneum is higher when there is an adnexal mass.

In the multivariate analysis, the type of conception was no longer statistically significant. The risk of a mass decreased with maternal age but increased with high hCG levels on D0.

## Discussion

According to the « Collège National des Gynécologues et Obstétriciens Français » (CNGOF), (Marret et al., 2016), MTX can be used as conservative treatment for uncomplicated EP with pre-therapeutic hCG level < 5000 IU/L, few symptoms and hemodynamic stability. Before injection, one should perform a blood test with kidney and liver function and complete blood count (Lesavre et al., 2015).

Contra-indications to MTX treatment include kidney or liver dysfunction, anemia, thrombopenia, leucopenia, acute pelvic or abdominal pain, hemodynamic instability, clinical or ultrasound suspicion of tubal rupture, hCG > 5000 IU/L, embryo heartbeat, unreliable patient, adnexal mass > 4 cm and/or important hemoperitoneum (Capmas et al., 2014; Cecchino et al., 2014; Lesavre et al., 2015; Fields et Hathaway, 2017). There is an increased risk of failure if a gestational sac is visible or if the patient has a history of medical or surgical treatment for an EP (Marret et al., 2016; Levin et al., 2019), though our results and those in Orozco et al. (2015) study showed that a previous EP did not influence the treatment outcome.

The aim of this study was to evaluate the efficacy of MTX treatment in EP including persisting PULs. We sought to identify several predictive factors of success or failure that would help to choose a treatment option.

We reported success after one injection in 67% of our patients. A second injection successfully treated 89% of the remaining patients. Treatment failure was observed in 20% of patients.

Various studies reported success of MTX in 65 to 95% of the cases (weighted mean 82%) (Capmas et al., 2014; Lesavre et al., 2015; Marret et al., 2016). Our results are consistent with these data with an 80% success rate (or 84% after exclusion of patients with hCG> 5000 IU/L). This rate is similar to the 78.5% success rate described in the retrospective study by Bonin et al. (2017), after one injection in 63.5% of the patients and after a second treatment in 73.2% of the remaining cases as well as in Levin et al. with a success rate of 62.7% after a single dose of MTX (Levin et al., 2019). Orozco et al. (2015), in their prospective study, demonstrated a global success rate of 88.1% while more recently Barbier et al. (2019) described a global success rate of 79.1%.

According to the findings of Cho et al. (2006), Helmy et al. (2014) and Bonin et al. (2017) clinical symptoms (metrorrhagia and pelvic pain) before treatment did not influence the treatment outcome. However, it is worth noting that patients with severe symptoms and/or those who are hemodynamically unstable are not good candidates for MTX treatment and were thus excluded from this study. Most patients included in our study had mild symptoms and 18% were asymptomatic.

Maternal age, gestational age and the type of pregnancy (spontaneous vs assisted) were not predictive factors either. Cho et al. (2006), Orozco et al. (2015) and Bonin et al. (2017) had similar results.

Concerning the TVS data, the mass size had no impact while the presence of a mass at TVS was a predictive factor of failure. This is consistent with Bonin et al. (2017) who showed that the presence of a hematosalpinx was predictive of failure (P = .03) with no influence of its size. However, this is potentially influenced by the fact that more than half (52%) of our EP were PULs. The success rate is indeed higher in case of PULs as their pre-treatment hCG levels are lower: the mean hCG level on D0 was 516.16 ± 501.88 IU/L and 2342.52 ± 2350.64 IU/L in case of PUL and adnexal mass respectively.

Incidence of EP within PUL is low (7-20%). Indeed, the majority of PULs would be spontaneous abortion of intrauterine pregnancy (Fields and Hathaway, 2017). Therefore, it is recommended to first monitor hCG levels (Capmas et al., 2014; Marret et al., 2016) as expectative management has proved to be effective in “less active” PULs. If the hCG level keeps increasing or is stable, risks of EP are higher (Fields and Hathaway, 2017). CNGOF recommends MTX in PULs when it lasts for more than 10 days in asymptomatic women or when hCG is higher than 2000 IU/L (Marret et al., 2016). In our study, 32 patients had a persisting PUL and 30 among them (93.75%) were successfully treated with MTX. Before treatment, an average of 10 days of observation was applied to evaluate hCG kinetics. There is no specific cut-off before giving MTX in PULs. However, this will be influenced by the speed at which the hCG level rises. MTX will be administrated after 3 to 4 consecutive increasing hCG levels. If hCG level decreases spontaneously, expectant management is recommended (Fields and Hathaway, 2017).

In the multivariate analysis, only hCG level on D0 remained significant and thus can be considered as the most important predictive factor. Although authors do not agree on a threshold (Marret et al., 2016), most articles do not recommend MTX when the hCG level on D0 is above 5000 IU/L (Lesavre et al., 2015). This is supported by the fact that in our cohort, all women with hCG > 4000 IU/L (n=4) needed surgery despite MTX injection, which suggests that a hCG level > 4000 IU/L is a major risk factor for treatment failure.

Several other studies tried to identify a threshold of hCG level for MTX treatment.

Rabischong et al. (2011) reported a success rate of 75.4% in a cohort of 419 patients and identified the threshold of 1,300 IU/L. Sagiv et al. (2012), studying a cohort of 238 women, demonstrated a success rate of only 59% when pretherapeutic hCG level was between 2000 and 3000 IU/L and suggested that MTX should not be used when hCG level is higher than 2000 IU/L. Helmy et al. (2014) studied a cohort of 198 patients and determined a hCG level threshold at 2121 IU/L (P < .001). Orozco et al. (2015) carried out a prospective study on 126 cases and described success for patients with hCG level < 1000 IU/L on D0. With a threshold of 2439 IU/L, our results are consistent with review articles that conclude that failure rate of medical treatment is higher when hCG level is above 2,000 IU/L (Marret et al., 2016).

In our study, low D4 and D7 hCG levels were predictive of success, confirming Bonin et al. (2017) and Barbier et al. (2019) results. However, Orozco et al. (2015) did not find that D4 hCG was predictive of treatment outcome. They suggested that a decrease of 19% between D4 and D7 was predictive of success whereas Barbier et al. (2019) and Levin et al. (2019) defined a decrease higher than 20% between D1 and D4 as an early indicator of success. In our study, a decrease lower than 15% between D4 and D7 was considered as an indication for a second injection, as described by Kirk et al. (2007).

Lastly, we found that the presence of pelvic pain, metrorrhagia and hemoperitoneum at TVS post- treatment were predictive factors of failure. Bonin et al. (2017) reported pain on D7 as predictive of failure but did not analyze the two other factors. Conversely, according to Rabischong et al. (2011) and Levin et al. (2019), pelvic pain after MTX injection is an expected symptom and is not always a sign of complication.

## Conclusion

MTX is a safe approach for the treatment of ectopic pregnancies. However, we showed that its efficacy decreases when pretreatment hCG levels are higher than 2439 IU/L and that it is ineffective when hCG is higher than 4000 IU/L. Low hCG level on D0, D4 and D7 are predictive factors of success. The presence of pain, metrorrhagia and hemoperitoneum in the days following the first MTX injection are predictive factors of failure.

On the one hand, these data are worth knowing when proposing treatment options to patients to inform them about the chances of success of MTX treatment. On the other hand, even though its efficacy is lower when hCG is > 2439 IU/L, MTX can still be considered, while informing patients about the high risk of failure.

This study brings additional information about the high efficacy of MTX treatment in the management of persisting PULs. As the incidence of PULs is increasing with assisted reproductive techniques, their management requires an intensive follow-up and a good knowledge concerning treatment options.

Whereas our results are consistent with previous articles, the limitations of the present study are its retrospective design and the relatively small number of patients. Further research on larger cohorts and with a prospective controlled study design would be helpful to eventually establish international guidelines.
